# Spectral correction for handheld optoacoustic imaging by means of near‐infrared optical tomography in reflection mode

**DOI:** 10.1002/jbio.201800112

**Published:** 2018-10-02

**Authors:** Leonie Ulrich, Linda Ahnen, Hidayet Günhan Akarçay, Salvador Sánchez Majos, Michael Jaeger, Kai Gerrit Held, Martin Wolf, Martin Frenz

**Affiliations:** ^1^ Institute of Applied Physics University of Bern Bern Switzerland; ^2^ Biomedical Optics Research Laboratory, Department of Neonatology University Hospital Zurich Zurich Switzerland

**Keywords:** blood oxygen saturation, fluence compensation, multimodal imaging, near‐infrared optical tomography, optoacoustic signal quantification, quantitative optoacoustic imaging, spectral correction

## Abstract

In vivo imaging of tissue/vasculature oxygen saturation levels is of prime interest in many clinical applications. To this end, the feasibility of combining two distinct and complementary imaging modalities is investigated: optoacoustics (OA) and near‐infrared optical tomography (NIROT), both operating noninvasively in reflection mode. Experiments were conducted on two optically heterogeneous phantoms mimicking tissue before and after the occurrence of a perturbation. OA imaging was used to resolve submillimetric vessel‐like optical absorbers at depths up to 25 mm, but with a spectral distortion in the OA signals. NIROT measurements were utilized to image perturbations in the background and to estimate the light fluence inside the phantoms at the wavelength pair (760 nm, 830 nm). This enabled the *spectral correction* of the vessel‐like absorbers' OA signals: the error in the ratio of the absorption coefficient at 830 nm to that at 760 nm was reduced from 60%‐150% to 10%‐20%. The results suggest that oxygen saturation (*SO*_*2*_) levels in arteries can be determined with <10% error and furthermore, that relative changes in vessels' *SO*_*2*_ can be monitored with even better accuracy. The outcome relies on a proper identification of the OA signals emanating from the studied vessels.

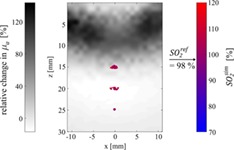

## Graphical abstract

Optoacoustics (OA) is combined with near‐infrared optical tomography (NIROT) in reflection mode to quantitatively image vasculature oxygen saturation (*SO*_*2*_) levels in phantoms containing vessels at depths up to 25 mm. Using NIROT to estimate the light fluence, the OA signals' spectral distortion was reduced from 60%‐150% to 10%‐20%. Results suggest that *SO*_*2*_ levels can be determined with <10% error and that temporal changes can be monitored with even better accuracy.

## 1 INTRODUCTION

The assessment of tissue and vasculature oxygenation is of paramount importance, not only for a better understanding and profiling of tissues with healthy physiology, but also for a more effective diagnosis of numerous pathologies through imaging hypoxia, hyperoxia or anoxia 1. The essential criteria for a reliable mapping of oxygenation in vivo include “noninvasiveness, adequate spatial and temporal resolution, ability to quantify oxygenation levels, low radiation exposure, good safety profile and widespread clinical availability” 2. It is difficult to fulfill these requirements with current imaging modalities 2, 3, 4, 5, optoacoustics (OA), however, has the potential to provide quantitative estimates of blood oxygenation in the (micro‐) vasculature. The OA signals recorded are proportional to the absorbed energy density, which can be assumed to be equal to the product of the optical absorption coefficient of the blood inside the vessels and the light fluence at the position of these vessels 6. By applying an appropriate spectral analysis, that is, by determining the fluence in the OA images acquired with different illumination wavelengths, it is possible to retrieve the shape of the blood's absorption spectrum and, subsequently, the absolute hemoglobin oxygen saturation (*SO*_*2*_) values inside these vessels 7. Accurately estimating the tissue's optical properties and the fluence is a complex task 8, which could, for example, be solved by using exogenous markers 9, 10 or additional modalities 11, 12.

A target application is the early detection of cerebral ischemia in preterm infants 13, 14. Cerebral ischemia is a key initiating factor for white matter injury 13, which has become the dominant pathology in the premature brain 15 and is a major reason for long‐term neurodevelopmental impairments 15, 16, 17. Reconstructing the optical properties of the infant brain by means of near‐infrared optical tomography (NIROT) has been investigated, based on the technical progress achieved in near‐infrared spectroscopy (NIRS) 18, 19, 20, 21, 22, 23, 24, 25. This article examines the feasibility of employing NIROT to estimate the light fluence at the positions of vessels imaged by OA. Using the detection of ischemia as a blueprint and following on from the work in refs. 12, 26, 27, 28, 29, we designed a hybrid OA/NIROT imaging system and conducted experiments on two phantoms representing simplified models of the infant brain. Both phantoms are composed of dye‐filled, submillimetric tubes embedded in a scattering background, which mimic part of the vasculature inside the white matter. One phantom also contains larger optical absorbers, designed to simulate a perturbation in the white matter (such as those caused by hemorrhages), changing the local fluence distribution and thereby provoking an alteration of the OA signals originating from the dye‐filled tubes. These two phantoms were used to model an infant brain at two successive points in time. Taking exclusively OA images it would not unambiguously be possible to decide without additional knowledge whether the alteration of the OA signals is due to changes in the *SO*_*2*_ level inside the vessels or due to the perturbation in the white matter. Our study demonstrates the advantage of combining OA with NIROT in, first, solving this ambiguity, and second, retrieving the ratio of the dye's absorption coefficients between the wavelength pair (*λ*_1_ = 760 nm, *λ*_2_ = 830 nm), with and without the simulated perturbation.

## 2 SPECTRAL CORRECTION OF 2D OA IMAGES BY MEANS OF NIROT: THEORY

**Figure 1:**
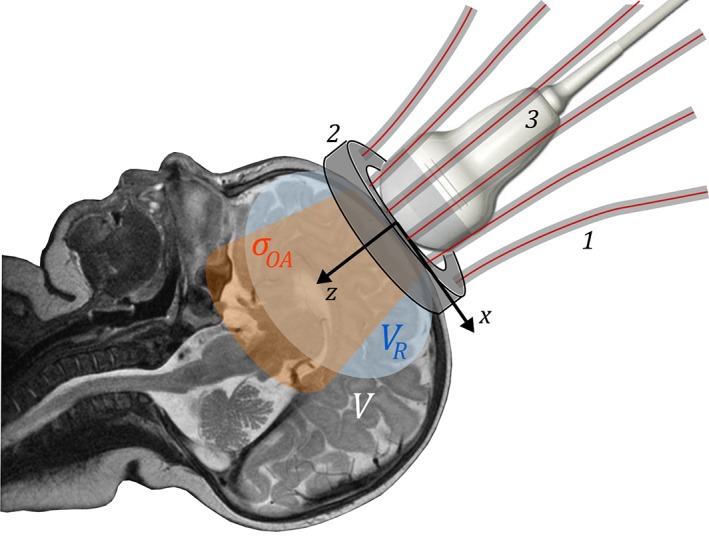
Schematic drawing of the combined OA/NIROT probe placed on an infant head, representing the volume *V* under investigation in the proposed clinical application. The NIROT fibers (1) are placed on a ring‐shaped holder (2) surrounding the fontanelle. The OA apparatus (3), composed of a US transducer and an illumination module (not shown here), is in the middle of this ring. The OA imaging plane *σ*_*OA*_ and a cross‐section of the volume *V*_*R*_ reconstructed with NIROT are shown in orange and blue, respectively

Figure 1 illustrates the geometry of the proposed combined OA/NIROT probe placed on an infant head: the fibers used for NIROT measurements are arranged on a ring‐shaped holder 30, in the center of which the OA apparatus (ultrasound [US] transducer and an illumination module) is placed. This combined probe is positioned such that the US transducer is aligned with the acoustically transparent fontanelle 31, 32. We model the tissue under investigation as a volume *V*, in which we distinguish a finite number of blood vessels, which are imaged by means of OA. To align with a pixelated OA image, we discretize *V* into a Cartesian grid of finite size and given resolution. To each voxel, or position **r** = (x, y, z), in the volume is assigned an absorption coefficient *μ*_*a*,*λ*_(**r**) and a reduced scattering coefficient $\mu_{s,\lambda }^{\prime }\left(r\right)$ for every wavelength *λ*. We assume that the reduced scattering coefficient $\mu_{s,\lambda }^{\prime }\left(r\right)$ does not significantly change across *V*, while heterogeneities in *μ*_*a*,*λ*_(**r**) can be significant, due to spatial variations in the concentration of the main chromophore hemoglobin 25. The aim of this study is to use OA imaging to retrieve the spectra of the absorption coefficients *μ*_*a*,*λ*_ at the positions of the submillimetric vessel‐like absorbers that cannot be resolved in NIROT reconstructions. The NIROT reconstructions are used to determine the light fluence Φ_*λ*_(**r**) arising from the OA illumination inside *V* which is used for the spectral correction of the OA signals.

### 2.1 OA signal generation and 2D spectral correction

In OA measurements, the tissue is illuminated by a laser source emitting light pulses that are approximated by a Dirac delta temporal profile. Thermoelastic conversion of light absorbed inside the tissue leads to the build‐up of an excess pressure *p*_*λ*_(**r**), which is the product of the quantities *μ*_*a*,*λ*_(**r**), Φ_*λ*_(**r**) and the Grüneisen parameter Γ(**r**) (thermoelastic conversion efficiency). The excess pressure acts as the source of a broadband transient ultrasound wave field. Spatially and temporally resolved detection of this wave field at the tissue surface using an array of US sensors allows the reconstruction of a quantity *S*_*λ*_(**r**). Under ideal conditions *S*_*λ*_(**r**) is equal to *p*_*λ*_(**r**) (ie, no frequency bandwidth limitation, detection area encompasses the sample). The proposed clinical setup, however, does not fulfill these ideal conditions, which has a strong influence on *S*_*λ*_(**r**): the limited aperture of linear array probes (typically around 10 mm length for transcranial probes) provides a high directional sensitivity within a thin two‐dimensional (2D) tissue slice (“OA imaging plane”, *σ*_*OA*_), defined by the direction of the line of the array elements and by the direction of maximum angular sensitivity. Due to the narrow angular sensitivity perpendicular to *σ*_*OA*_, the OA signal amplitude depends on the angle of optically absorbing structures relative to the imaging plane 33. This results in a scaling of the signal amplitude with an a priori unknown factor *G*(**r**). In addition, the limited frequency bandwidth of the US transducer (typically of the order of 80% of the center frequency, *f*_0_) results in a spatially dependent image point spread function *PSF*(**r**^′^, **r**) that acts like a bandpass filter for spatial variations of optical absorption on the length scale of the acoustic wavelength at *f*_0_. The reconstructed image *S*_*λ*_(**r**) can therefore be expressed as

(1) $$S_{\lambda }\left(r\right)\propto \int_{\sigma_{\mathrm{OA}}}G\left(r^{\prime }\right)\mu_{a,\lambda }\left(r^{\prime }\right)\Phi_{\lambda }\left(r^{\prime }\right)\;\mathrm{PSF}\left(r^{\prime },r\right)\;dr^{\prime }$$

Owing to the dependence of *S*_*λ*_(**r**) on the spatial orientation of the blood vessels and on the band‐limited *PSF*, the absolute value of *S*_*λ*_(**r**) does not allow for a quantitative reconstruction of *μ*_*a*,*λ*_(**r**)Φ_*λ*_(**r**). However, the *SO*_*2*_ can be recovered by analyzing the wavelength‐dependent variation of *S*_*λ*_(**r**) ∝ *μ*_*a*,*λ*_(**r**)Φ_*λ*_(**r**) 34, provided that Φ_*λ*_(**r**) is known. To enable this analysis, we identify, for each vessel, a *support*, which is defined as a set of pixels in which the signal values *S*_*λ*_(**r**) are determined by the optical absorption inside that vessel. The support can be any set of pixels that simultaneously fulfills the following two conditions: (1) all terms in the product *G*(**r**)*μ*_*a*,*λ*_(**r**)Φ_*λ*_(**r**) in Eq. 1 are constant within the intersection of the vessel's actual cross‐section with the support, and (2) the support area does not interfere with that of an adjacent vessel.

As the spatial profile of the signal values *S*_*λ*_ which originate from a vessel does not depend on the wavelength, the ratio of *μ*_*a*,*λ*_Φ_*λ*_ (which approximates the absorbed energy density within the vessel) at different optical wavelengths *λ*_1_ and *λ*_2_ can be determined quantitatively from the pixelwise ratio of *S*_*λ*_:

(2) $$R_{\mathrm{OA}}=\frac{S_{\lambda 2}}{S_{\lambda 1}}=\frac{\mu_{a,\lambda 2}\cdot \Phi_{\lambda 2}}{\mu_{a,\lambda 1}\cdot \Phi_{\lambda 1}}.$$

*R*_*OA*_, which is only meaningful within the support, is central to our analysis as it leads to the determination of the ratio of absorption coefficients at the two wavelengths, $R_{\mu_{a}}=\mu_{a,\lambda 2}/\mu_{a,\lambda 1}$ (reflecting the relative concentrations of oxyhemoglobin [O_2_Hb] and deoxyhemoglobin [HHb]), and consequently to the determination of the *SO*_*2*_ value in the vessel. Although the ratio *R*_*OA*_ is determined pixelwise, there is no one‐to‐one correspondence between the spatial distribution of the pixels in the support and the size/shape of the actual vessel cross‐section, due to the convolution of p_λ_(**r**) with the *PSF* (including side lobes and limited view artifacts).* The pixel values within the support reflect a set of statistical realizations of *R*_OA_ originating from the vessel.

The *SO*_*2*_ in a vessel can be evaluated based on a statistical analysis of *R*_*OA*_ over the support, but its determination requires information on the wavelength‐dependent fluence Φ_*λ*_ at the position of that vessel within the OA imaging plane. For each pixel in the support, we calculate a spectrally corrected OA signal ratio ${\tilde{R}}_{\mathrm{OA}}$:

(3) $${\tilde{R}}_{\mathrm{OA}}=\frac{{\tilde{S}}_{\lambda 2}}{{\tilde{S}}_{\lambda 1}}=\frac{R_{\mathrm{OA}}}{R_{\Phi }}=R_{\mu_{a}},$$

with ${\tilde{S}}_{\lambda }=S_{\lambda }/\Phi_{\lambda }$ the corrected OA signal and *R*_Φ_ = Φ_*λ*2_/Φ_*λ*1_ the pixelwise ratio of fluences at wavelengths *λ*_1_ and *λ*_2_. The wavelength‐dependent local fluence Φ_*λ*_, which depends on the tissue geometry and composition, that is, on the spatial distribution of optical properties within *V* 8, 35, cannot be measured directly, but it can be estimated using NIROT.

### 2.2 NIROT and NIRFAST: principle, calibration and regularization scheme

In NIROT measurements, the tissue volume *V* is probed with a given number of light sources and detectors, all of which are coupled via optical fibers to the tissue's surface. We work here with “frequency‐domain NIROT,” that is, the light sources are intensity‐modulated with a sinusoidal profile. Each combination of a source and a detector fiber (source‐detector pair) leads to the detection of a time‐dependent signal, characterized by a DC intensity, AC amplitude and phase of the sine wave, which carry complementary information on the optical properties inside the probed “banana‐shaped” region 36. The measurements are used to solve an inverse problem, which, for every wavelength, reconstructs a pair of optical properties $\left(\mu_{a,\lambda },\mu_{s,\lambda }^{\prime }\right)$ for every voxel of a volume *V*_*R*_ 37.

Practical limitations in the number of source‐detector pairs together with the diffusive propagation of light in tissue make the NIROT inverse problem underdetermined and ill‐posed. Regularization reduces the space of possible solutions by favoring spatial distributions of optical properties that are measurable by the given geometry of source‐detector pairs. These are typically characterized by slow spatial variations and low spatial resolution. Despite this constraint, in practical applications NIROT can produce tomographic images with resolutions in the order of 5 mm 38, 39.

To solve the NIROT inverse problem, we use NIRFAST, a software package developed at Dartmouth University 40. This Matlab‐based code solves the light diffusion equation on a tetrahedral mesh by the finite element (FEM) approach, and relies on Tikhonov regularization to constrain the space of solutions. If the measurements at the tissue surface are denoted by *ψ*^*M*^ (phase shifts and logarithms of AC amplitude decays) and the calculated data using the forward solver by *ψ*^*C*^, then the standard Tikhonov minimization function *χ*^2^ for the reconstruction of the optical properties *μ* (for a chosen initial guess *μ*_0_) is given by

(4) $$\chi^{2}=\min_{\mu }\left\{\sum_{i=1}^{\mathrm{NM}}{\left(\delta \psi_{i}\right)}^{2}+\gamma \sum_{j=1}^{\mathrm{NN}}{\left(\mu_{j}-\mu_{0}\right)}^{2}\right\},$$

where $\delta \psi_{i}=\psi_{i}^{M}-\psi_{i}^{C}$, *NM* is the total number of measurements, *NN* is the number of FEM nodes (the unknowns) and *γ* is the Tikhonov regularization parameter. Optical properties are updated iteratively according to:

(5) $$\delta \mu ={\left(J^{T}J+2\mathrm{\gamma I}\right)}^{-1}J^{T}\delta \psi ,$$

where *I* is the identity matrix and *J* the Jacobian matrix containing derivatives of logarithms of AC amplitude decays and phase shifts for each source‐detector pair with respect to the optical properties in each node of the mesh. *δψ* is the data‐model misfit at the current iteration and *δμ* is the difference between optical properties at the current iteration and the previous one. In NIRFAST, the iteration is terminated when the relative change of updated optical properties is smaller than a convergence cutoff, which we kept at 2%.

One problem in the reconstruction is the lack of a unique set of solutions: *μ*_*a*,*λ*_ and $\mu_{s,\lambda }^{\prime }$ have an interchangeable influence on the simulated data, so that many different configurations of optical properties can explain the recorded measurements 25. Here, this effect is partially reduced by implementing the assumption that spatial variations in $\mu_{s,\lambda }^{\prime }$ are much lower than variations in *μ*_*a*,*λ*_, which allows the choice of a regularization strength, determined empirically, that is 100 times stronger for $\mu_{s,\lambda }^{\prime }$ than for *μ*_*a*,*λ*_. In this case, the choice of the initial guess for $\mu_{s,\lambda }^{\prime }$ is especially relevant.

The accuracy of the reconstruction depends on careful pre‐calibration of the NIROT system. This could be done using a reference phantom with known optical properties, where amplitude factors and phase offsets are determined 41. In addition, the mismatch between the discrete FEM space and the physical reality leads to meshing artifacts in the reconstructed properties as well as the simulated light fluence. In the present study, we have opted for a novel approach (further developing methods described in refs. 41, 42), to account simultaneously for amplitude factors, phase offsets and meshing artifacts. Optical properties are reconstructed based on a newly defined Δ*ψ*:

(6) $$\Delta \psi =\left(\psi^{M}-\psi^{M,\mathrm{ref}}\right)-\left(\psi^{C}-\psi^{C,\mathrm{ref}}\right)$$

where the differences between measurements taken on the studied object and those acquired on an optically homogeneous reference phantom are considered. *ψ*^*M*^ is the data measured on the studied object, *ψ*^*M*,*ref*^ is the data measured on the reference phantom, *ψ*^*C*^ is the forward simulated data based on the current guess and *ψ*^*C*,*ref*^ is the simulated data of the reference phantom according to its known optical properties. This approach serves as a “custom calibration” procedure and does not require a prior determination of amplitude factors and phase offsets, as they cancel out via subtraction. Similarly, a noticeable reduction of meshing artifacts can be achieved if the same mesh is used for both the studied object and the reference phantom, whose geometry and optical properties are taken to be similar to those of the studied object. The choice of the reference phantom additionally minimizes residual reconstruction artifacts stemming from imperfect light coupling.

Finally, the reconstructed optical properties are fed into NIRFAST to perform forward simulations of the light fluence (in the plane defined by the OA imaging plane *σ*_*OA*_), induced by the OA illumination. The spectral correction for the OA signals originating from the vessels is then carried out according to Eq. 3: the uncorrected OA signal ratio *R*_OA_ is pixelwise divided by the fluence ratio *R*_Φ_ (the fluence is resampled from the tetrahedral mesh to a Cartesian grid, with the finer pixel resolution of the OA image), resulting in the corrected OA signal ratio ${\tilde{R}}_{\mathrm{OA}}$ in the vessel supports.

## 3 EXPERIMENTAL TISSUE MODEL

### 3.1 Phantom design and geometry

Two cuboid phantoms were built (referred to as phantom 1 and phantom 2), to roughly model the infant brain at two different points in time, before and after the occurrence of a perturbation in its optical properties, for example, due to hemorrhages. The actual shape and structure of the infant head is not accounted for and the influence of boundaries (other than at *z* = 0) on the light propagation is avoided by choosing appropriately large phantoms. However, we aimed to use materials that, as far as possible, optically mimic an infant's brain 43.

Phantom 1 is comprised of an optically homogeneous, strongly scattering liquid background material. Inside this background medium, three identical tubes with a *μ*_*a*_ value higher than that of the background were inserted to mimic blood vessels. These vessel‐like inclusions were aligned vertically, in parallel to the *y*‐axis, at a depth of *z* = 15 mm, *z* = 20 mm and *z* = 25 mm, respectively. Phantom 2 is quasi‐identical to phantom 1, with the difference being that it includes two supplementary optical heterogeneities in the form of larger cylinders that have $\mu_{s}^{\prime }$ values similar to that of the background medium, while their *μ*_*a*_ value at 760 nm is higher than that of the background. These cylinders are intended to model the aforementioned perturbation in the tissue surrounding the vessels. They have a diameter of 6 mm and a height of 10 mm, and are positioned with their central axis at (*x*, *y*) = (+7.5 mm, −6 mm) and (*x*, *y*) = (−7.5 mm, 0 mm), respectively, and with their top surface at *z* = 5 mm.

Phantoms 1 and 2 were probed with OA to retrieve the (spectrally distorted) OA signal ratio *R*_*OA*_ of the three vessel‐like tubes (signal at 830 nm divided by signal at 760 nm). NIROT measurements and reconstructions were performed to determine the light fluences in both phantoms, to subsequently calculate the corrected OA signal ratios ${\tilde{R}}_{\mathrm{OA}}$. In the two phantoms, the background material's optical properties are such that *R*_*OA*_ varies in depth and the difference between *R*_*OA*_ and ${\tilde{R}}_{\mathrm{OA}}$ is noticeable for all three tubes. In phantom 2, the positions and absorption coefficients of the cylinders were chosen to accentuate the spectral distortion already present in phantom 1, and to yield an exploitable contrast in the NIROT reconstructions.

The accuracy of the spectral correction is assessed by comparing the retrieved corrected OA signal ratios ${\tilde{R}}_{\mathrm{OA}}$ to the reference value $R_{\mu_{a}}$: according to Eq. 3, in the case of a perfect spectral correction, these ratios should be equal. To evaluate the accuracy of the fluence distributions determined with NIROT/NIRFAST used to perform the correction, they were compared to reference fluence distributions. In phantom 1, the light fluence can be calculated by the light diffusion approximation Φ^*D*^(**r**) for a semi‐infinite, homogeneous medium 44, 45:

(7) $$\Phi^{D}\left(r\right)\propto \frac{z}{d^{3}}\left(1-d\cdot {\left(3\mu_{a}\mu_{s}^{\prime }\right)}^{1/2}\right)e^{-d\cdot {\left(3\mu_{a}\mu_{s}^{\prime }\right)}^{1/2}},$$

where *d* is the distance between the OA illumination point on the phantom's surface and the position **r**. Phantom 1 served as a reference to (1) demonstrate the performance attainable with NIROT/NIRFAST, as the reconstructions for such a volume are expected to yield reliable results without any a priori knowledge, and to (2) experimentally establish the efficiency of quantitative OA imaging with our system. The reference fluence distribution in phantom 2 was calculated by Monte Carlo simulations† 46, 47, 48. Calculating the reference fluences for both phantoms was possible by optically characterizing all individual volume components independently at both wavelengths.

### 3.2 Phantom preparation and optical properties of its individual components

The background medium consists of a lipid solution, which is considered to be a fairly realistic model of biological tissues and is easy to handle and reproduce 49, 50. It was prepared by diluting 4.5 vol% of a fat emulsion (SMOFlipid 20%, Fresenius Kabi, Switzerland) with de‐ionized water and Naphthol Green (Naphthol Green B for microscopy, Sigma‐Aldrich, Switzerland) to produce a total volume of 7000 mL, with a fat concentration of 0.9 vol% and a dye concentration of 1.75 μg mL^−1^.

The vessels were implemented with polyethylene tubes (Smiths Medical, Minneapolis, Minnesota, inner diameter 0.28 mm, wall thickness 0.16 mm), filled with a solution of Indocyanine Green (ICG) dye (IR‐125, Laser Grade, ACROS Organics, Geel, Belgium). The ICG dye was dissolved in ethanol (Ethanol p.a., Sigma‐Aldrich, Switzerland) resulting in a concentration of 5 μg mL^−1^ to attain a spectrally varying optical absorption in the same order of magnitude as the absorption of hemoglobin at 760 and 830 nm 51, 52. The low dye concentration used in this study and the choice of ethanol as the solvent guaranteed that both fluorescence and bleaching effects were negligible and did not affect the measured OA signals.

Based on the known absorption coefficients of the ICG solution, a reference value for the aforementioned $R_{\mu_{a}}$ was calculated for all vessels, by dividing the *μ*_*a*_ at 830 nm by the *μ*_*a*_ at 760 nm: $R_{\mu_{a}}=0.31$. For the fabrication of the two absorbing cylinders in phantom 2, transparent silicone (SILPURAN 2420 A/B, Wacker Chemie AG, Munich, Germany) was colored with ELASTOSIL RAL blue 5022 (Wacker Chemie AG, Munich, Germany).

The optical properties of all phantom components are summarized in Table 1.

**Table 1:** Optical properties of the individual phantom components at 760 nm and 830 nm

Sub‐volume	Wavelength (nm)	*μ*_*a*_ (mm^−1^)	$\mu_{s}^{\prime }$ (mm^−1^)	$R_{\mu_{a}}$
Background medium (fat emulsion)	760 830	0.0066 (±1%) 0.0048 (±2%)	0.91 (±2%) 0.82 (±2%)	— —
Vessels (ICG solution)	760 830	0.22 (±3%) 0.068 (±3%)	— —	0.31 (±4%)
Cylinders (dyed silicone)	760 830	0.16 (±10%) 0.0075 (±10%)	0.64 (±10%) 0.55 (±10%)	— —

The background medium was characterized with time of flight measurements using the method described in ref. 53, the absorption spectrum of the ICG solution was determined using a commercial spectrophotometer (PerkinElmer Inc., Waltham, Massachusetts), and the optical properties of the silicone cylinders were measured with a commercial NIRS device (Imagent, ISS Inc., Champaign, Illinois). Measurement uncertainties are given in brackets.

## 4 EXPERIMENTAL SETUP, DATA ACQUISITION AND EVALUATION PROCEDURES

### 4.1 Setup description and importance of intrinsic co‐registration

The measurement setup (see Figure 3) mimics the proposed clinical system shown in Figure 1, however, there are noticeable differences with respect to both the probing geometry and the acquisition procedure. The ring‐shaped NIROT probe is replaced by a linear sensor; however, the circular geometry is attained de facto, by rotating this sensor. As for US detection, a large aperture vascular probe is used instead of a transcranial probe. Both modalities are sequentially operated in reflection geometry. The OA and NIROT probes are attached to a common probe holder. This ensures an intrinsic geometric co‐registration of two‐dimensional OA images and the NIROT volumetric reconstructions, which is crucial to performing a pixel‐based spectral correction. The common probe holder was designed such that the rotation axis of the NIROT probe coincides with the center axis of the OA probe, along the *z*‐axis.

For the OA imaging system, we employed a diode‐pumped Q‐switched Nd:YAG laser with integrated optical parametric oscillator (Spitlight DPSS OPO, InnoLas Laser GmbH, Krailling, Germany), providing laser pulses with 10 nanoseconds length at a rate of 100 Hz, in the wavelength range of 650 to 900 nm. The light pulses were coupled into a 1 mm core multimode fiber (Thorlabs, Newton, New Jersey) with a numerical aperture of *n* = 0.39. The fiber was inclined by 15° with respect to the *z*‐axis. The wavelength‐ and time‐dependent laser pulse energy provided by the OPO system was monitored by reflecting a small fraction of the light onto a pyroelectric energy sensor before coupling into the fiber. The recorded average output pulse energy at the fiber tip was ~5 mJ. For OA signal acquisition, we used a linear array ultrasound probe with *f*_0_ = 5 MHz center frequency featuring 128 elements with a total aperture of 38 mm at a Δ*x* = 0.298 mm element pitch (ATL L7‐4, Philips N.V., Netherlands), connected to a research ultrasound system (V 1‐64, Verasonics, Kirkland, Washington). The center of the ultrasound probe aperture was positioned at the origin of the lab coordinate frame and the illumination fiber tip at (*x*, *y*, *z*) = (0 mm, −18 mm, −1 mm).

NIROT measurements were performed with a commercially available NIRS device (Imagent, ISS Inc., Champaign, llinois). This device employs photomultiplier tubes as detectors and laser diodes as sources emitting at 760 and 830 nm, modulated with a frequency of 110 MHz. We created a rigid fiber holder to ensure a reliable positioning of the fiber tips. Four illumination fibers were placed along a line at 2, 2.5, 3 and 3.5 cm from a detector fiber (see Figure 3B). These source‐detector distances represent the distances available within the area to be probed on the infant head.

### 4.2 Data acquisition and analysis

#### 4.2.1 NIROT: measurement protocol, choice of the initial guess

For the NIROT measurements, we ensured that the source and detector fiber tips were slightly dipped into the liquid phantom, to achieve a stable coupling and avoid light piping. Tomographic data were acquired by rotating the sensor in 30 steps of Δ*α* = 12^°^, covering 360°. This resulted in 30 × 4 = 120 source‐detector pairs, that is, measurements *NM*, in total.

**Figure 2:**
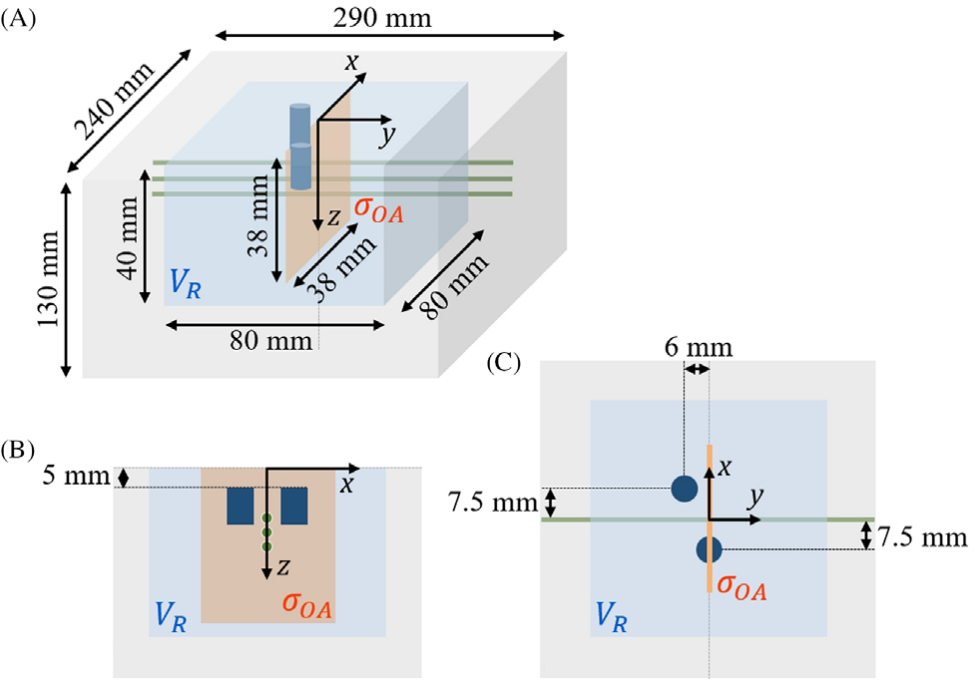
Schematic drawing (not to scale) of phantoms 1 and 2 (A), together with side and top view (B and C). Both phantoms consist of subvolumes characterized by different absorption and reduced scattering coefficients: the background medium (in gray) and three vessel‐mimicking inclusions (in green). The two optical heterogeneities (in dark blue) model a perturbation in phantom 2 but are absent in phantom 1. As in Figure 1, the OA imaging plane *σ*_*OA*_ and the volume *V*_*R*_ reconstructed by means of NIROT are shown in orange and blue, respectively

The sizes of the reconstructed volumes are smaller (−40 mm ≤ *x* ≤ 40 mm, −40 mm ≤ *y* ≤ 40 mm, 0 ≤ *z* ≤ 40 mm) than the actual dimensions of the phantoms (see Figure 2), in order to keep the computational burden within reasonable limits, but still large enough that boundary effects did not influence the fluence calculated at the positions of the vessel‐like inclusions. The node separation distance of 0.6 mm was a compromise between the computational costs and the spatial resolution achievable, given the number of measurements 54.

A key step during the reconstructions is the choice of the initial guesses 55, 56. We opted for an approach that does not rely on a priori assumptions and that is applicable in a clinical scenario 41. For each wavelength, a pair of initial guesses for *μ*_*a*_ and $\mu_{s}^{\prime }$ was retrieved by fitting, for each rotation angle *α*, the recorded data set to the frequency‐domain light diffusion model for a homogeneous, semi‐infinite medium 57, and by averaging the fit results over *α*. It should be noted that the choice of the initial guesses strongly influences the outcome of the reconstructions, since any deviations of the initial guesses from the true values will not be corrected in regions where the sensitivity of the probing source‐detector pairs is low. In a final step, the light fluences were calculated from the reconstructed optical properties for both wavelengths and both phantoms, by means of NIRFAST‐based forward simulations.

#### 4.2.2 OA: measurement protocol and signal segmentation

**Figure 3:**
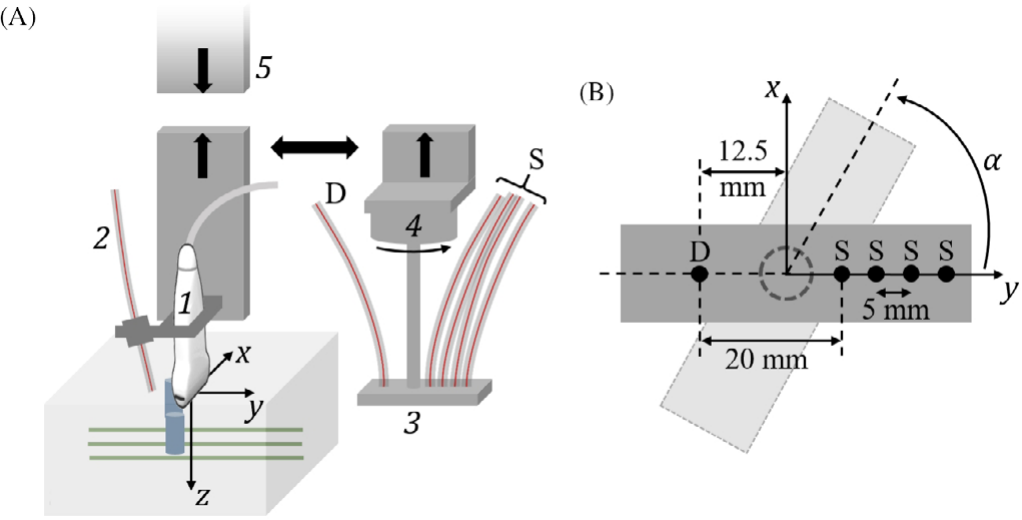
A, Schematic drawing (not to scale) of the experimental setup. The OA probe consists of a linear array transducer (1) and an illumination fiber (2). The NIROT probe features four fibers for illumination (S) and one fiber for detection (D), fixed to a linear fiber holder (3), which is attached to a rotational stage (4). OA and NIROT probes are sequentially mounted on a common probe holder (5) for an intrinsic registration between the two modalities. B, Cross‐section of the NIROT linear sensor in the *x*, *y*‐plane, showing illumination (S) and detection (D) fibers. Tomographic data were acquired by rotating the sensor stepwise around the *z*‐axis by an angle *α* ranging from 0° to 360°

Figure 3A shows a sketch of the position of the phantom relative to the OA probe, with the embedded vessels aligned perpendicularly to the imaging plane *σ*_*OA*_, so that the linear transducer array provides maximum sensitivity for the vessel signals. The surface of the probe aperture was slightly dipped in the liquid phantom.

Datasets were acquired by averaging over 600 consecutive light pulses. This acquisition was repeated for 10 wavelengths, ranging from 750 to 840 nm, with a step size of 10 nm, forming one acquisition sequence. This number of wavelengths was required for image segmentation and was a tradeoff between acquisition time and segmentation quality. In total, we repeated the acquisition sequence for five times. A frequency‐domain algorithm 58, 59 was used to reconstruct images with a spatial resolution of 149 by 74 μm in the *x*‐ and *z*‐direction, respectively.

In section 2, we identified *S*_*λ*_ as the output of the linear OA reconstruction algorithm (see Eq. 1), that is, the radio‐frequency (rf‐)mode image. As a result of the limited bandwidth of the probe, the amplitude in the rf‐mode image oscillates, along *z*, around zero mean with a period determined by the center frequency of the ultrasound receiver. Hence, calculating the OA signal ratio *R*_*OA*_ (signal at 830 nm divided by signal at 760 nm) from this rf‐mode image as indicated in Eq. 2 has a significant disadvantage: the spatial oscillation leads to zero‐crossings where the ratio *R*_*OA*_ is not defined or is highly inaccurate in presence of noise or tissue motion. To avoid this problem, we redefine *S*_*λ*_ as being the envelope of the rf‐mode image, which is a purely positive function. Even though calculating the envelope constitutes a nonlinear operation, Eq. 2 is still valid.

An integral part of the quantitative analysis of the OA signal ratio *R*_*OA*_ according to Eqs. 2 and 3 is the identification/segmentation of the supports corresponding to the vessels in *S*_*λ*_. The segmentation was based on the assumption that the OA signal originating from a vessel is correlated across different wavelengths, while the background signal is uncorrelated. For each pixel, spectral correlation was quantified by averaging, over consecutive wavelength pairs (*λ*_*i*_, *λ*_*i* + 1_), the product of the analytic signal at *λ*_*i*_ with the complex conjugate at *λ*_*i* + 1_. The supports were identified by setting a threshold for the spectral correlation. To determine this threshold, we had to make a tradeoff between preserving a statistically meaningful number of pixels in the supports, while excluding pixels where the signal is governed by noise. We empirically established that this is achieved if the support of the vessel at 25 mm depth contains 25 pixels.

## 5 RESULTS

### 5.1 Uncorrected OA image ratios: quantifying spectral distortion

**Figure 4:**
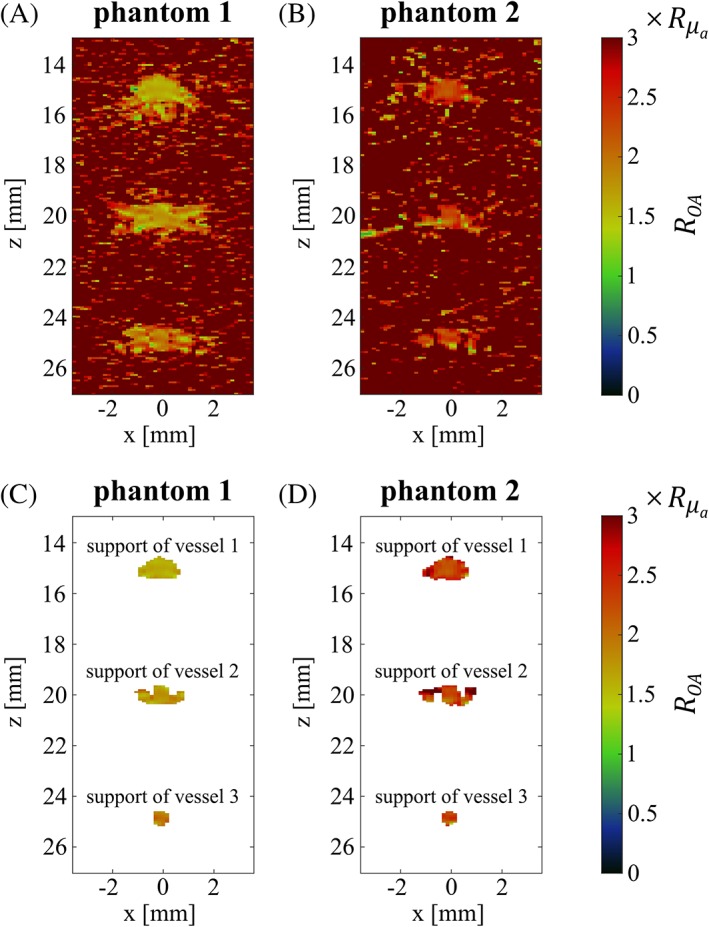
Uncorrected OA signal ratio *R*_*OA*_ in units of the reference $R_{\mu_{a}}$ for phantom 1 (A) and phantom 2 (B) shown in the selected ROI. The same data is displayed in (C) for phantom 1 and (D) for phantom 2, after segmentation of the supports

Figure 4A,B show the uncorrected OA signal ratio *R*_*OA*_ compared to the reference ratio $R_{\mu_{a}}$ for both phantoms averaged in each case over the five acquisitions. For better visualization, the data is shown in a region of interest (ROI) covering the supports. The cylindrical inclusions in phantom 2, located outside the ROI, could not be imaged due to the limited bandwidth of the US probe. Within the supports, the pixel values quantify the spectral distortion (and in the absence of any distortion, the OA signal ratio *R*_*OA*_ is expected to be equal to the reference ratio $R_{\mu_{a}}$). The supports can qualitatively be distinguished from the noisy background, but without a clear delineation. The segmented OA signal ratios are given in Figure 4C,D, for phantoms 1 and 2, respectively. Figure 4 clearly reveals that the supports have noncircular, irregular geometries and that their areas exceed the actual area of the vessel‐mimicking tubes, due to the influence of the *PSF*. The lower signal‐to‐noise ratio (SNR) at a depth of 25 mm results in a weaker spectral correlation, and thus, in a smaller support for the third vessel. It is also apparent that values of the OA signal ratio are nonuniform across each support and deviate from the reference. For phantom 1, the spectral distortion increases with depth, with the OA signal ratio ranging on average from $∼1.6\times R_{\mu_{a}}$ for the vessel at 15 mm to $∼1.9\times R_{\mu_{a}}$ for that at 25 mm, despite the fact that in all vessels, the dye concentration and absorption spectrum is identical. In phantom 2, the distortion is even stronger, with the OA signal ratio being in the order of $2\times R_{\mu_{a}}$ to $2.5\times R_{\mu_{a}}$. In an in vivo monitoring scenario where the same infant brain is probed at two different points in time, it would be difficult without NIROT to infer whether the difference in spectral distortion observed between the two points in time is the result of a change in the vessels' *SO*_2_ levels, or that of a perturbation occurring in the surrounding tissue.

### 5.2 NIROT reconstructions of optical properties

The initial guesses for *μ*_*a*_ and $\mu_{s}^{\prime }$ determined according to the technique described in section 4.2.1 are given in Table 2, for both wavelengths and both phantoms. There was no discernible difference between the NIROT measurements (phase shifts and logarithms of AC amplitude decays) on phantom 1 and those on the homogeneous reference phantom, that is, as anticipated our NIROT system was not sensitive to the presence of the three vessels. Under these conditions, the retrieved initial guesses for phantom 1 are close to the background material's reference optical properties, with ~1% to 3% inaccuracy at 760 nm and ~7% to 15% inaccuracy at 830 nm. It can be seen that the presence of the additional cylindrical inclusions in phantom 2 yields different initial guess values. The sets of initial guesses were included in the reconstruction algorithm, to determine the absorption and reduced scattering coefficient for every voxel, for both wavelengths and both phantoms.

**Table 2:** Initial guesses of optical properties for phantoms 1 and 2, at 760 and 830 nm

Phantom	Wavelength (nm)	Initial guess for *μ*_*a*_ (mm^−1^)	Initial guess for $\mu_{s}^{\prime }$ (mm^−1^)
1	760	0.0068 (+3%)	0.90 (−1%)
	830	0.0055 (+15%)	0.76 (−7%)
2	760	0.0072 (+9%)	1.05 (+15%)
	830	0.0057 (+19%)	0.78 (−6%)

The relative deviations (in %) from the reference optical properties of the background medium (see Table 1) are given in brackets.

**Figure 5:**
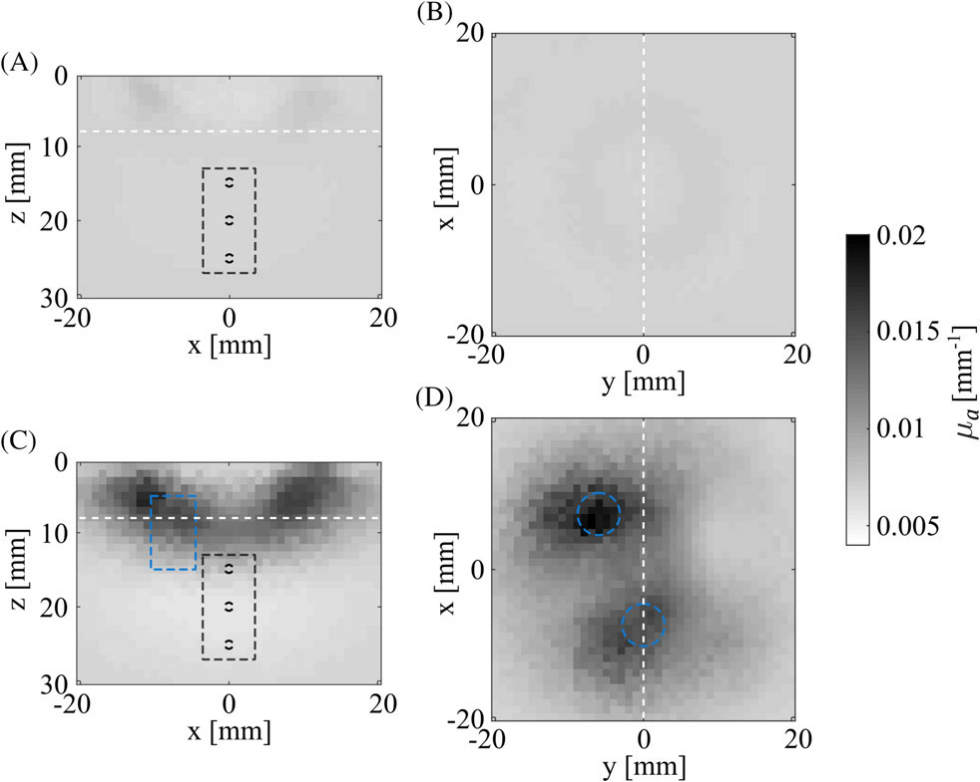
Spatial distributions of the reconstructed *μ*_*a*_ values at 760 nm along the OA imaging plane for phantom 1 (A) and phantom 2 (C) and in the *x*, *y*‐plane at *z* = 8 mm for phantom 1 (B) and phantom 2 (D). Black‐dashed lines: vessel‐like tubes and ROI. Blue‐dashed lines: absorbing cylindrical inclusions. White‐dashed lines in A and C indicate the planes shown in B and D and vice versa

Two‐dimensional cross‐sections of *μ*_*a*_ through phantom 1 at 760 nm are given in Figure 5A (along the plane defined by the OA imaging plane) and Figure 5B (along a plane of constant depth at *z* = 8 mm). Black‐dashed lines indicate the positions of the vessels and the ROI. The three vessels are not visible and the distribution of optical properties appears to be quasi‐uniform across the phantom. The reconstructed *μ*_*a*_ differs by <0.5% from the initial guesses and diverges from the background's reference *μ*_*a*_ on average by ~3% at 760 nm and ~15% at 830 nm. These results are quite remarkable, given that no a priori knowledge was employed in the NIROT reconstructions. The deviations from the reference values give a measure of the accuracy that can be expected from NIROT reconstructions with the chosen experimental setting.

Similarly, Figure 5C,D shows *μ*_*a*_ in cross‐sections through phantom 2. As in phantom 1, the vessels are not detected, but the larger cylinders (which have a strong absorption at 760 nm compared to the background) are qualitatively imaged. Closer examination of Figure 5C,D reveals that the contrast in the reconstructed *μ*_*a*_ extends to regions outside the actual cylinders' locations and that the absolute values of *μ*_*a*_ are considerably lower than the cylinders' real absorption coefficient (the maximum reconstructed *μ*_*a*_ still underestimates the reference *μ*_*a*_ of the cylinder by a factor ~5): this combined effect is a consequence of the ill‐posedness and underdetermination of the reconstruction problem 60. Across phantom 2, the reconstructed *μ*_*a*_ values differ, on average, from the reference values by ~6% at 760 nm. At 830 nm, the cylindrical inclusions are not visible in the reconstruction (not shown here), since their optical properties are comparable to that of the background. At this wavelength, the reconstructed *μ*_*a*_ values deviate by about ~15% from the background's known absorption coefficient. As opposed to the results obtained in phantom 1, the reconstructed *μ*_*a*_ values deviate noticeably from the initial guesses (8% at 760 nm and 4% at 830 nm). Due to the strong regularization implemented into the reconstruction procedure, $\mu_{s}^{\prime }$ values for both phantoms globally differ less than 1% from the initial guess (also not shown here): the inaccuracy of $\mu_{s}^{\prime }$ is given by that of the initial guess (<15%, according to Table 2).

From a diagnostic viewpoint, the visibility of the absorbing cylinders in the reconstructions shown in Figure 5C,D confirms the presence of a perturbation in the tissue. This provides valuable complementary information to the OA data given in section 5.1: in vivo, this would suggest that the changes in the OA signal ratio between the two points in time represented by phantoms 1 and 2 might be due to this perturbation and not to actual variations in the blood's *SO*_*2*_. This hypothesis can be validated by examining the corrected OA data with and without the presence of the perturbation. The quality of the correction depends on the estimation of the light fluence inside the volume, and in the following we are interested in inspecting the effect of the quantitative errors in reconstructed optical properties on the fluences determined for both phantoms.

### 5.3 Fluence ratios obtained with NIRFAST

To validate the performance of NIROT/NIRFAST, we compared the fluence distributions determined by NIROT/NIRFAST to calculated reference fluence distributions. In lieu of comparing the fluence distributions themselves, we compared the respective fluence ratios $R_{\Phi }^{\mathrm{NF}}$ (the fluence at 830 nm divided by the fluence at 760 nm), since this ratio directly determines the quality of the spectral correction of the OA images.

**Figure 6:**
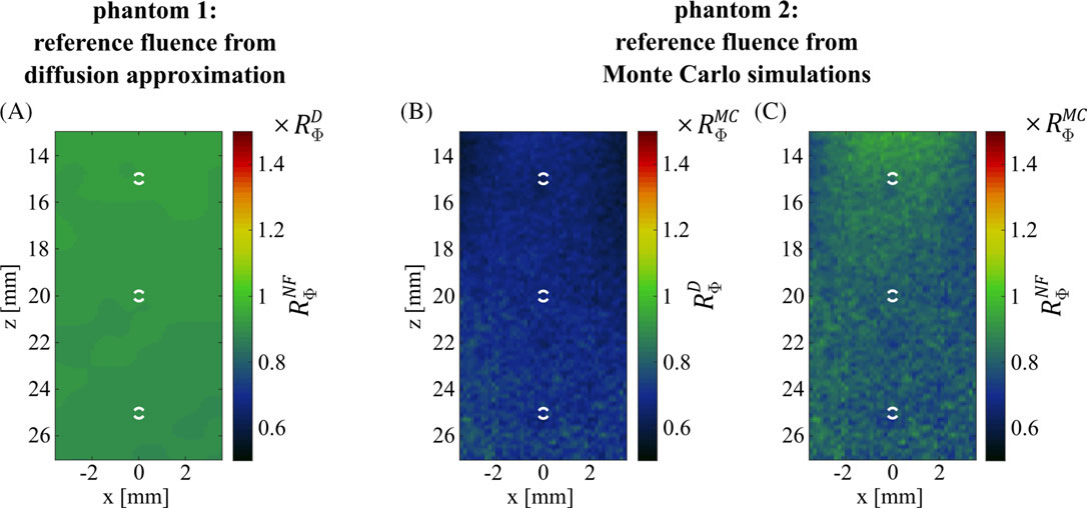
Phantom 1: Fluence ratio $R_{\Phi }^{\mathrm{NF}}$ obtained with NIRFAST in units of the reference fluence ratio $R_{\Phi }^{D}$, calculated based on the analytical diffusion approximation (A). Phantom 2: fluence ratio $R_{\Phi }^{D}$ obtained with the analytical diffusion approximation (neglecting the cylindrical inclusions) in units of $R_{\Phi }^{\mathrm{MC}}$ from Monte Carlo simulations (B) and fluence ratio $R_{\Phi }^{\mathrm{NF}}$ from NIRFAST, also in units of $R_{\Phi }^{\mathrm{MC}}$ (C). All images are confined to the ROI; white circles indicate actual cross‐sections of the vessels

Figure 6A shows the fluence ratio $R_{\Phi }^{\mathrm{NF}}$ obtained with NIRFAST for phantom 1, compared to the reference fluence ratio $R_{\Phi }^{D}$ obtained by applying the analytical diffusion approximation. Very good agreement can be observed: $R_{\Phi }^{\mathrm{NF}}$ varies only between $0.88\times R_{\Phi }^{D}$ and $0.94\times R_{\Phi }^{D}$ across the whole ROI (a perfect agreement would result in $R_{\Phi }^{\mathrm{NF}}$ being equal to $R_{\Phi }^{D}$). The analytical diffusion approximation is known to provide a reliable fluence estimation in phantom 1, and as such the accordance between $R_{\Phi }^{\mathrm{NF}}$ and $R_{\Phi }^{D}$ in this phantom gives a measure of the accuracy to be expected from NIROT/NIRFAST under favorable conditions.

Figure 6B compares the fluence ratio $R_{\Phi }^{D}$ obtained from the analytical diffusion approximation with the reference fluence ratio $R_{\Phi }^{\mathrm{MC}}$ for phantom 2, determined with Monte Carlo simulations. $R_{\Phi }^{D}$ is identical to the reference fluence in Figure 6A (for phantom 1) and was thus determined assuming a homogeneous medium, while $R_{\Phi }^{\mathrm{MC}}$ takes the absorbing cylinders into account. The substantial differences between $R_{\Phi }^{D}$ and $R_{\Phi }^{\mathrm{MC}}$ underline that the presence of the cylindrical inclusions cannot be neglected. By comparing Figure 6B to Figure 6C (the latter compares $R_{\Phi }^{\mathrm{NF}}$ with the reference from Monte Carlo simulations), it becomes apparent that there is a much better agreement between the fluence distribution provided by NIRFAST and the reference obtained by Monte Carlo simulations than there is between the fluence calculated with the diffusion approximation and the reference from Monte Carlo simulations. This underlines the advantage of using NIROT to estimate the fluence distribution in the presence of a perturbation, over methods that assume a homogeneity of the background medium. For phantom 2, $R_{\Phi }^{\mathrm{NF}}$ ranges from approximately $0.71\times R_{\Phi }^{\mathrm{MC}}$ to $0.92\times R_{\Phi }^{\mathrm{MC}}$ across the ROI and is thus quantitatively not as close to the reference as the one for phantom 1, which can be attributed to the higher degree of complexity involved.

### 5.4 Spectrally corrected OA signal ratios: correction quality and relevance of NIROT

The uncorrected OA signal ratios *R*_*OA*_ (see Figure 4) have shown that signals in all vessel supports are affected by spectral distortions and that these are stronger in phantom 2 than in phantom 1. NIROT reconstructions and light fluence calculations have revealed that the differences in the distortions between the phantoms may be explained by the perturbation in phantom 2. To conclude our results, we calculate the corrected OA signal ratios ${\tilde{R}}_{\mathrm{OA}}$, from the uncorrected ratios *R*_*OA*_ and the fluence ratios *R*_Φ_.

**Figure 7:**
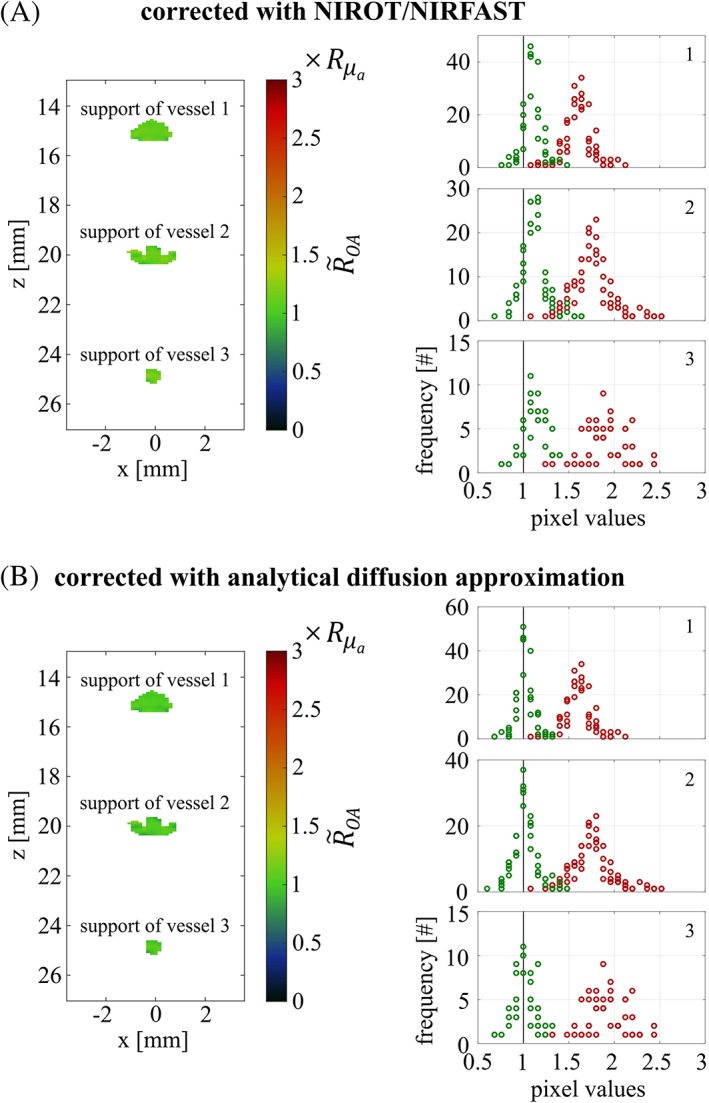
OA signal ratio ${\tilde{R}}_{\mathrm{OA}}$ for phantom 1 corrected with NIROT/NIRFAST (A) and corrected based on the analytical diffusion approximation (B), in units of the reference ratio $R_{\mu_{a}}$, together with histogrammed pixel values within the supports, before (red) and after (green) fluence correction. In the subplots showing the histograms, numbers refer to the vessel index and perfect agreement with the reference is indicated with a black line

The results after spectral correction for phantom 1 are shown in Figure 7 by displaying the corrected OA signal ratio ${\tilde{R}}_{\mathrm{OA}}$ (averaged over five acquisitions) in units of the reference ratio $R_{\mu_{a}}$. In Figure 7A, the correction has been performed using NIROT/NIRFAST, whereas in Figure 7B, the correction was undertaken with the fluence distribution calculated with the analytical diffusion approximation. By comparing these images with those shown in Figure 4C, the necessity for spectral correction becomes evident (${\tilde{R}}_{\mathrm{OA}}$ almost equals $R_{\mu_{a}}$). Comparing Figure 7A and B reveals that spectral correction for phantom 1 with NIROT is as good as with the analytical diffusion approximation. To quantify the spectral correction, while accounting for the nonuniformity within the supports, we plotted histograms of the pixel values within the supports before (red) and after (green) correction for all five acquisition sequences and determined the deviation of the histograms' peaks from the reference. Before correction, the peak positions of the histograms lie between $1.6\times R_{\mu_{a}}$ and $1.9\times R_{\mu_{a}}$ for the three vessels, while after correction the peak positions are between $1.1\times R_{\mu_{a}}$ and $1.2\times R_{\mu_{a}}$ for the correction with NIROT/NIRFAST and at $\leq 1.05\times R_{\mu_{a}}$ for the correction with the analytical diffusion approximation.

**Figure 8:**
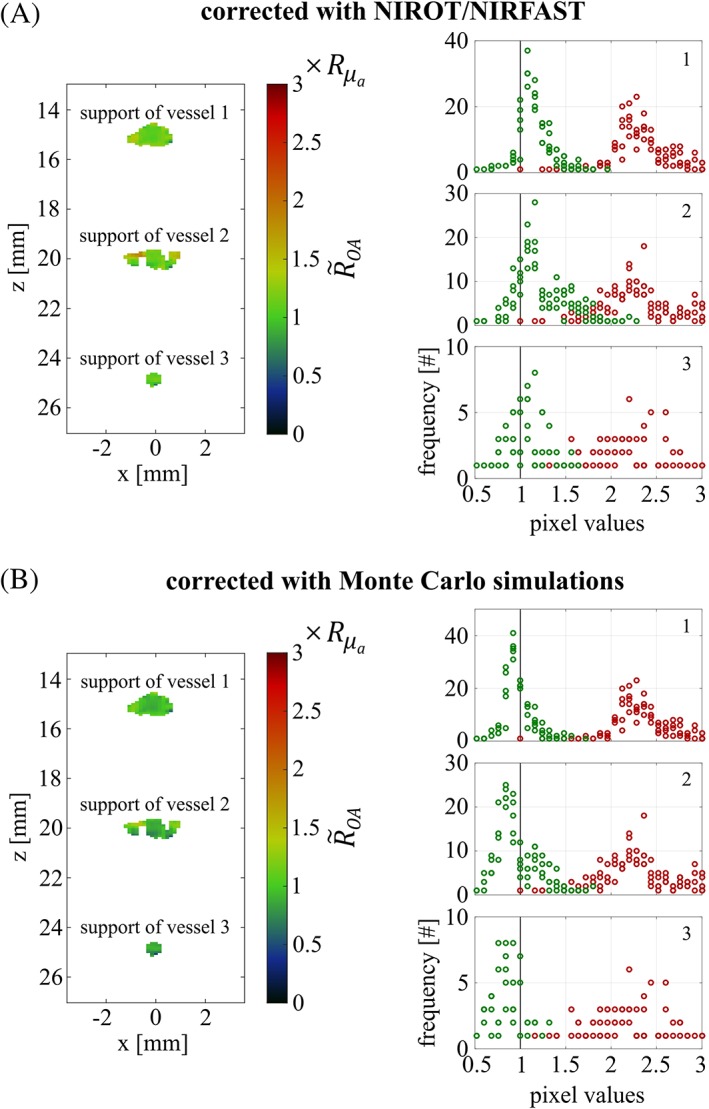
OA signal ratio ${\tilde{R}}_{\mathrm{OA}}$ for phantom 2 corrected with NIROT/NIRFAST (A) and corrected based on Monte Carlo simulations (B), in units of the reference $R_{\mu_{a}}$, together with histogrammed pixel values within the supports, before (red) and after (green) correction. In the subplots showing the histograms, numbers refer to the vessel index and perfect agreement with the reference is indicated with a black line

Figure 8A,B shows the results for phantom 2 after spectral correction performed with NIROT/NIRFAST and based on Monte Carlo simulations. The comparison between Figure 8A and B reveals that both methods perform equally well. The peak positions of the histograms showing the corrected OA image ratio ${\tilde{R}}_{\mathrm{OA}}$ are at $<1.2\times R_{\mu_{a}}$ and $>0.8\times R_{\mu_{a}}$, respectively.

It is worth stressing how effectively NIROT corrects the OA signals for all three supports, both in phantom 1 and phantom 2. NIROT accounts for the spatial and spectral variations of the light fluence inside the probed volume of phantom 2 despite the uncertainties in the reconstruction of the cylindrical inclusions' positions and optical properties. In our assumed in vivo situation, the combined results from OA and NIROT would clearly assert that the differences obtained between the two different points in time are not due to changes in the vessels' *SO*_*2*_, but due to a perturbation in the surrounding tissue.

## 6 INTERPRETATION OF THE SPECTRAL CORRECTION RESULTS

This study has demonstrated that under favorable conditions (ie, calibration/probing procedure and choice of initial guesses), NIROT constitutes a clinically viable modality to complement OA for the spectral correction of vessel signals in a combined reflection‐mode handheld setup. Second, we have shown its value for understanding temporal variations of the OA signals, via monitoring of tissue surrounding the vessels. Furthermore, we have demonstrated that the quantitative evaluation of OA images relies on the identification of the supports representing individual blood vessels.

### 6.1 On the shape/size and the pixelwise nonuniformity of signals originating from vessels in 2D OA reconstructions

In OA imaging, the *PSF* depends not only on the system, but also on the a priori unknown spatial distribution of acoustic properties, which causes the variations in size and shape of the segmented supports corresponding to the different vessels imaged in our experiments. These variations, however, do not pose a problem: rather than the spatial characteristics of the supports, it is the statistics of the pixel values therein that are of interest for our quantitative analysis. As can be seen in Figure 4, the pixel values within the supports have a nonuniform distribution although the two conditions stated in section 2.1 (that have to be fulfilled by the supports) indicate that the OA signal ratio *R*_*OA*_ must be constant across the supports. A violation of the first condition seems implausible, as the small size of the vessels and the low concentration of the absorbing dye used make nonuniformities in the absorbed energy density within the vessels very unlikely 6. The second condition is equally fulfilled, since the individual supports do not overlap. The pixelwise spectral correction (ie, dividing the OA image ratio by the 2D light fluence ratio across the full support area) also cannot be responsible for the nonuniformity. Indeed, the SD of the fluence ratios $R_{\Phi }^{\mathrm{NF}}$ across the supports was <1% for all vessels and both phantoms.

**Figure 9:**
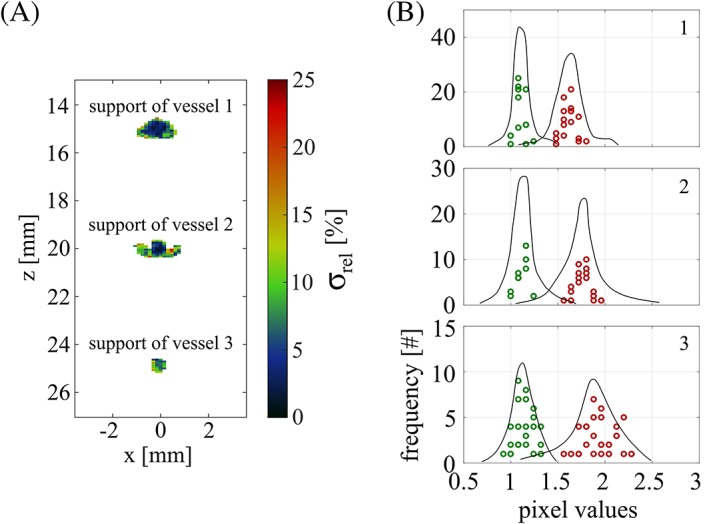
A, Pixelwise relative SD *σ*_*rel*_ of *R*_*OA*_ across the five acquisition sequences for phantom 1. B, Histogrammed pixel values showing uncorrected OA signal ratios *R*_*OA*_ (red) and corrected OA signal ratios ${\tilde{R}}_{\mathrm{OA}}$ (green), both in units of the reference, in the central parts of the supports for phantom 1, overlaid by the histogram contours from Figure 7A (black lines)

Upon thorough examination of all the data we believe that the nonuniformity is rather due to motion of the vessels caused, for example, by convection inside the liquid background medium or by the repeated flushing of the ICG dye solution into the vessel‐mimicking tubes. Simulations indicated that a displacement of only 50 μm during the acquisition time would explain the observed nonuniformity. In agreement with this hypothesis, we obtain random fluctuations of *R*_*OA*_ mainly toward the profile tails, so that the SD is higher at the border of the support. This is illustrated by Figure 9A, where we show the pixelwise SD of the OA signal ratio *R*_*OA*_ across the five acquisition sequences (carried out over the course of ~10 minutes) for phantom 1, reflecting temporal fluctuations in *R*_*OA*_. Restricting the quantitative analysis to the central parts of the supports reduces the width of the *R*_*OA*_ and ${\tilde{R}}_{\mathrm{OA}}$ histograms (see Figure 9B) but keeps the peak positions of the histograms unchanged. The nonuniformity across the supports should, therefore, not be considered as a source of error. Still, we acknowledge it as being central and inherent to the analysis of OA signals originating from blood vessels, as motion might happen in vivo even more than in a well‐controlled phantom experiment.

We propose that given the nonuniformity across the support, the chosen histogram technique guarantees a robust quantitative analysis: the peak position intrinsically reflects *R*_*OA*_ values stemming from pixels that are most reliable within the support.

### 6.2 Evaluating the quality and reproducibility of the OA measurements

Results obtained with phantom 1 can be exploited to determine the accuracy of the spectral correction achieved with our OA system. As shown in Figure 7B, we identified an error of ≤2% for the first two vessels and an error of ≤5% for the third vessel, based on the peak positions in the histograms of the corrected OA signal ratio ${\tilde{R}}_{\mathrm{OA}}$ values. To verify the reproducibility of these results, we repeated the entirety of the measurements five times (ie, five times the five acquisition sequences described in section 4.2.2) on phantom 1, which was produced anew for each repetition. The independently retrieved corrected OA signal ratios ${\tilde{R}}_{\mathrm{OA}}$ (corrected with $R_{\Phi }^{D}$) are on average $∼1.05\times R_{\mu_{a}}$ across the supports, with a SD of ~4%. Although these results indicate an excellent, reproducible spectral correction, some sources of systematic errors limit the accuracy of the outcomes. The deviation of the corrected OA signal ratio ${\tilde{R}}_{\mathrm{OA}}$ from $R_{\mu_{a}}$ partially stems from the uncertainties in the optical properties of the phantom's background material and the ICG absorber (~2% and ~3%, respectively: see Table 1), which in turn translate into uncertainties in the reference fluence ratio $R_{\Phi }^{D}$ and the corrected OA signal ratios ${\tilde{R}}_{\mathrm{OA}}$ (error in the order of 4%‐5%), as well as in the reference ratio $R_{\mu_{a}}$ (error approximately 4%). Moreover, in the course of our experimental work, we established that OA measurements can be influenced by spectral and temporal fluctuations in the laser pulse energy that are not accounted for in the monitoring method described in section 4.1. Temporal variations in the ratio between the energy measured at the sample and in the reference path added an uncertainty of ~1% to 3% to the OA signal ratio.

### 6.3 Investigating the clinical applicability of our technique

#### 6.3.1 Calculating exemplary SO_2_ maps from corrected OA image ratios: translation of errors

In order to determine the quality of the spectral correction of the OA images with NIROT, we have compared the uncorrected and corrected OA signal ratio, *R*_*OA*_ and ${\tilde{R}}_{\mathrm{OA}}$, across the supports to the reference ratio $R_{\mu_{a}}$, calculated from the known absorption spectrum of the ICG solution used to mimic blood. In an in vivo experiment, where actual blood vessels are to be imaged, the clinically relevant parameter is the oxygen saturation level *SO*_2_ within a vessel, which can be calculated from the ratio of absorption coefficients between two wavelengths *λ*_1_ and *λ*_2_, that is, the corrected OA ratios ${\tilde{R}}_{\mathrm{OA}}$, according to

(8) $${\mathrm{SO}}_{2}=\frac{{ɛ}_{\lambda 2}^{\mathrm{HHb}}-{ɛ}_{\lambda 1}^{\mathrm{HHb}}{\tilde{R}}_{\mathrm{OA}}}{{ɛ}_{\lambda 2}^{\mathrm{HHb}}-{ɛ}_{\lambda 2}^{O_{2}\mathrm{Hb}}-\left[{ɛ}_{\lambda 1}^{\mathrm{HHb}}-{ɛ}_{\lambda 1}^{O_{2}\mathrm{Hb}}\right]{\tilde{R}}_{\mathrm{OA}}}.$$

**Figure 10:**
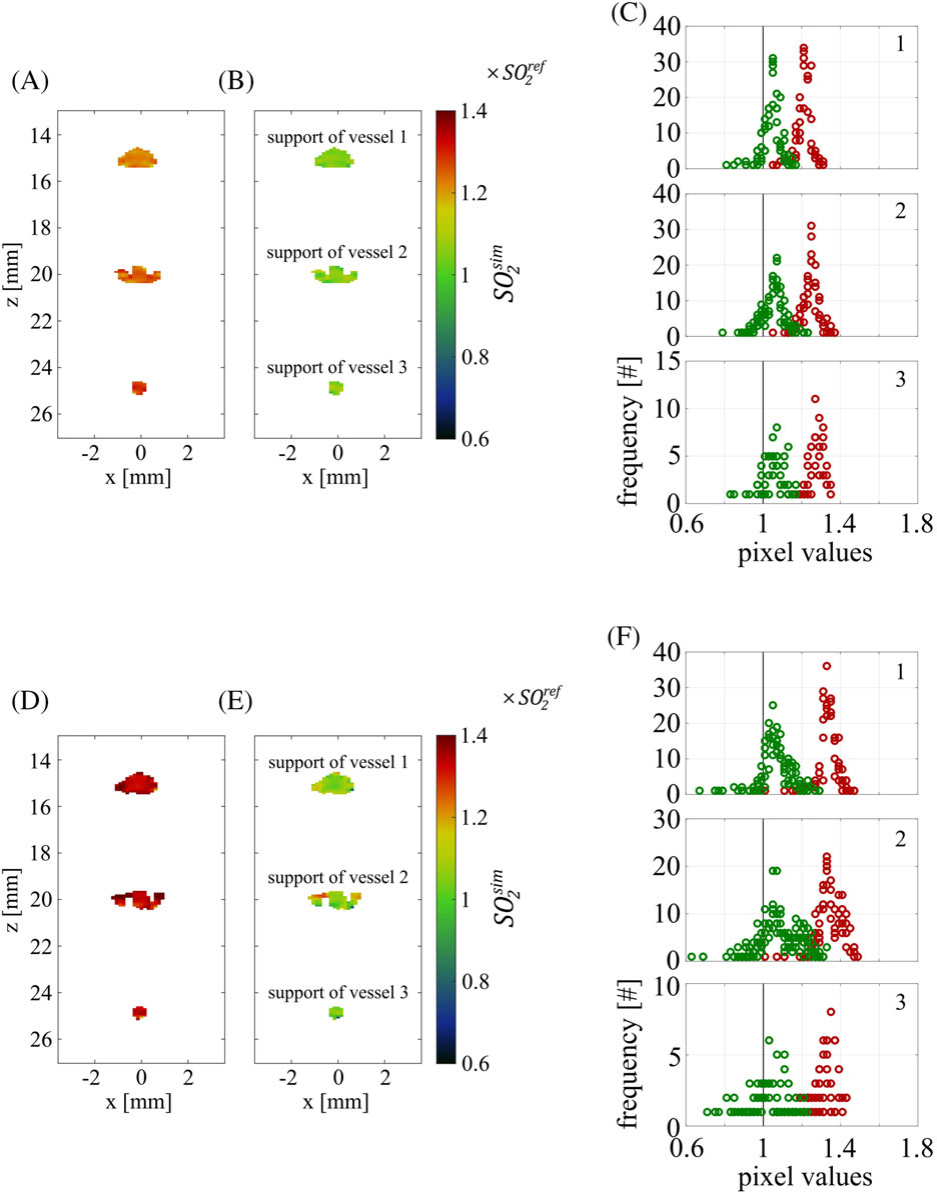
Simulated *SO*_*2*_ levels (${\mathrm{SO}}_{2}^{\mathrm{sim}}$) are shown in units of the reference *SO*_*2*_ level (${\mathrm{SO}}_{2}^{\mathrm{ref}}$) before (A, D) and after (B, E) correction for phantoms 1 and 2, respectively. The histogrammed pixel values are displayed in C and F

To understand the clinical significance of the experimental results, we assumed that the tubes were filled with blood instead of the ICG dye solution. Based on the absorption spectrum of blood with an *SO*_*2*_ value of ${\mathrm{SO}}_{2}^{\mathrm{ref}}=98\%$ 52, we generated artificial, spectrally distorted and corrected OA data, by keeping the same relative errors as the ones observed in section 5 between uncorrected or corrected OA signal ratios and the reference. This time, instead of plotting uncorrected and corrected OA signal ratios in units of the reference across the supports for both phantoms, we plot the *SO*_*2*_ values obtained from the artificial OA data (${\mathrm{SO}}_{2}^{\mathrm{sim}}$) in units of the reference *SO*_*2*_ value (${\mathrm{SO}}_{2}^{\mathrm{ref}})$. This is shown in Figure 10, before (A and D) and after correction (B and E). The uncorrected *SO*_*2*_ values were calculated by replacing in Eq. 8 the corrected OA signal ratio ${\tilde{R}}_{\mathrm{OA}}$ by the uncorrected ratio *R*_*OA*_. In Figure 10C,F, as in the representation used in section 5, we plot histograms of the *SO*_*2*_ values across the supports. A perfect correction would result in ${\mathrm{SO}}_{2}^{\mathrm{sim}}$ values equal to ${\mathrm{SO}}_{2}^{\mathrm{ref}}$: before correction, the peak positions of the ${\mathrm{SO}}_{2}^{\mathrm{sim}}$ values are in the range of $1.2\times {\mathrm{SO}}_{2}^{\mathrm{ref}}$ to $1.4\times {\mathrm{SO}}_{2}^{\mathrm{ref}}$; after spectral correction, the values are at $1.05\times {\mathrm{SO}}_{2}^{\mathrm{ref}}$ to $1.1\times {\mathrm{SO}}_{2}^{\mathrm{ref}}$. This indicates that, after correction, absolute *SO*_*2*_ values can be estimated with <10% error for both phantoms. Furthermore, our results suggest that the relative change in *SO*_*2*_ between two points in time can be measured with much higher accuracy than the absolute value. This is seen in Figure 10, as no deviation in peak positions between C and F is visible after correction.

**Figure 11:**
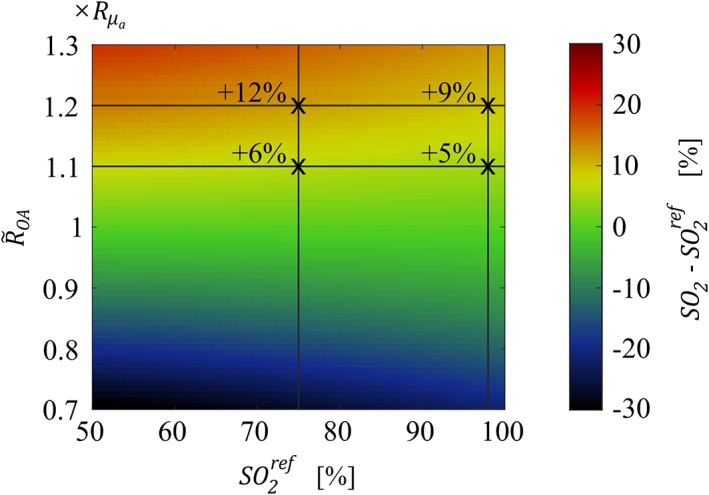
The difference between *SO*_*2*_ levels calculated according to Eq. 8 (*SO*_*2*_) and reference *SO*_*2*_ levels (${\mathrm{SO}}_{2}^{\mathrm{ref}}$) (on absolute scale) are shown as a function of the agreement of the corrected OA signal ratio ${\tilde{R}}_{\mathrm{OA}}$ with the reference $R_{\mu_{a}}$ and as a function of ${\mathrm{SO}}_{2}^{\mathrm{ref}}$. Crosses indicate the differences for ${\tilde{R}}_{\mathrm{OA}}=1.1\times R_{\mu_{a}}$ and ${\tilde{R}}_{\mathrm{OA}}=1.2\times R_{\mu_{a}}$ at a reference *SO*_*2*_ value of 98% (arterial blood) and 75% (venous blood)

The error in determining *SO*_*2*_ levels does not only depend on the quality of the spectral correction but also on the reference value ${\mathrm{SO}}_{2}^{\mathrm{ref}}$, as the propagation of errors in the corrected OA signal ratios ${\tilde{R}}_{\mathrm{OA}}$ into *SO*_*2*_ varies with ${\mathrm{SO}}_{2}^{\mathrm{ref}}$. Figure 11 illustrates this dependency for different inaccuracies in the spectral correction. Applying the inaccuracies obtained in our phantom experiments, which correspond to ${\tilde{R}}_{\mathrm{OA}}$ values of $1.1\times R_{\mu_{a}}$ to $1.2\times R_{\mu_{a}}$, an absolute error of around 5% to 9% for arterial *SO*_*2*_ levels (${\mathrm{SO}}_{2}^{\mathrm{ref}}$~98%), and an absolute error of around 6% to 12% for venous blood (${\mathrm{SO}}_{2}^{\mathrm{ref}}$~75%) can be expected. Moreover, the error in the estimation of *SO*_*2*_ levels depends on the selection of wavelengths 61. In this study, the wavelength pair (*λ*_1_ = 760 nm, *λ*_2_ = 830 nm) was given by the commercial NIRS/NIROT apparatus we worked with. In the future, the selection of a larger set of wavelengths could improve the *SO*_*2*_ accuracy to potentially meet that achieved by other non‐invasive techniques that are currently in clinical use but lack spatially resolved information 62.

#### 6.3.2 Further prospects: completing SO_2_ maps on a larger scale by using NIROT reconstructions

**Figure 12:**
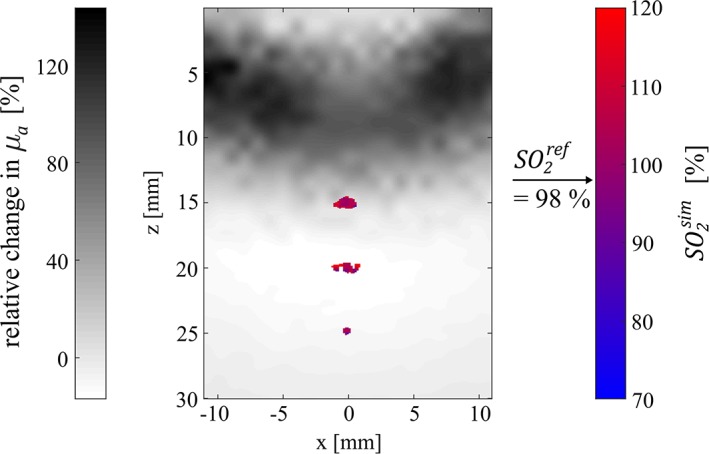
Hybrid OA/NIROT imaging: *SO*_*2*_ maps within submillimetric vessels are completed in the background by the relative change in *μ*_*a*_, at 760 nm, from phantom 1 to phantom 2. The chosen reference *SO*_*2*_ level in the vessels is 98%

From a diagnostic standpoint, it is of great interest to simultaneously visualize the (de)correlations between *SO*_*2*_ levels in the vasculature and the physiological/functional changes in the surrounding tissue. This is illustrated in Figure 12, where we have reconstructed a hybrid OA/NIROT image: here, Figure 10E has been superimposed onto a NIROT image displaying the relative changes in *μ*_*a*_ at 760 nm (obtained from the reconstructions presented in Figure 5). Such a hybrid OA/NIROT image not only displays spatially resolved *SO*_*2*_ levels in the vessels, but also reveals the occurrence of a perturbation in the background between the time points matching phantoms 1 and 2.

Considering the fact that a hybrid OA/NIROT imaging system additionally offers the possibility to use classical echo ultrasound imaging, this multimodal system will provide anatomical tissue information as well as quantitative *SO*_*2*_ levels in single vessels at an imaging depth of 2 to 3 cm. Such an imaging device is of high diagnostic value not only for the proposed application in neonatology but also, for example, in oncology.

## 7 SUMMARY AND OUTLOOK

In this study, we implemented a hybrid OA/NIROT system operated in reflection mode to assess to what extent NIROT can be used to correct spectrally distorted OA signals emanating from vessel‐like structures. The setup and experiments were designed and conducted to comply with the prerequisites for a reliable in vivo mapping of oxygenation in the brain of preterm infants. Our results, obtained from measurements on optically heterogeneous phantoms, indicate that a clinically promising spectral correction is feasible: in the OA data, the errors in the ratio of the blood‐mimicking dye's absorption coefficient at 830 nm to that at 760 nm decreased from >100% to <20%, for vessel‐like tubes positioned at depths ≤25 mm. This means that, if we were to assess *SO*_*2*_ values in arteries, the estimation error could be reduced from >30% to <10%. This error could potentially be further reduced by exploiting the full capacity of the hybrid OA/US/NIROT modality functioning on the basis of a mutual feedback system, where OA and US images are used to provide NIROT reconstructions with a priori knowledge 63, 64, 65, 66. Such mutual feedback could also be used to facilitate the interpretation of the quantitative data delivered in vivo by OA, NIROT and US. Our phantom experiments show that NIROT reconstructions could help in corroborating results obtained with OA, as they offer monitoring of the background tissue to detect the occurrence of a perturbation over time. In a similar manner, clinically meaningful *SO*_*2*_ values retrieved with OA could serve as a quality measure of the NIROT reconstructions.

Moreover, in quantitative OA imaging the proper estimation of the light fluence is often cited as the main challenge, but we demonstrated that the proper identification and interpretation of the vessel signals within the reconstructed OA images deserves equal attention. We therefore recommend the segmentation of the support corresponding to an individual vessel, as well as an appropriate statistical analysis to account for the nonuniformity of the pixel values across this support.

Despite the excellent results obtained in this phantom study it is still difficult to predict whether this hybrid imaging modality will perform as well in vivo where further optimization strategies tailored to the high complexity and heterogeneity of the target tissue might be needed 25, 54. Future steps will include the construction of the prototype sketched in Figure 1 to examine the applicability of our multi‐modal system for the early detection of cerebral ischemia in preterm infants. In particular, effects induced by the nontrivial geometry of the head or by shadowing due to densely distributed vessels/capillaries are yet to be further investigated.

## AUTHOR BIOGRAPHIES

Please see Supporting Information online.

## Supporting information


**Author Biographies**

## NOTES

*
Note that from a practical viewpoint, grating lobes should not be included into the support, as they occur at a location “far away” from the vessel and it will be difficult to establish a correspondence with a specific vessel.

†
Monte Carlo simulations were performed with a spatial resolution of 1/6 mm and by generating 10^10^ photon paths.
