# Supplementary material for: Spectral correction for handheld optoacoustic imaging by means of near‐infrared optical tomography in reflection mode
Source: J Biophotonics. 2018 Oct 2;12(1):e201800112. doi: 10.1002/jbio.201800112 (PMC7065640; doi:10.1002/jbio.201800112)
Supplement: Supplementary file 1 — Author Biographies [file JBIO-12-e201800112-s001.docx]

| 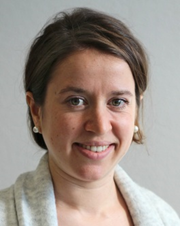 | **Leonie Ulrich** received her BSc in Physics, in 2013, from the University of Zürich, Zürich, Switzerland, and her MSc in Physics, in 2016, from the University of Heidelberg, Germany. Currently she is a PhD student at the Institute of Applied Physics, University of Bern, Switzerland. Her research focuses on quantitative optoacoustic imaging and light propagation in scattering media. |
| --- | --- |
| 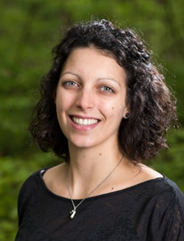 | **Linda Ahnen** is currently a PhD student at the Biomedical Optics Research Laboratory of the University Hospital in Zürich, Zürich, Switzerland. She graduated with a Diploma in Physics from the Technical University of Munich, Germany. Her research is focused on near-infrared optical tomography applied to determine the brain's oxygenation of preterm babies. |
| 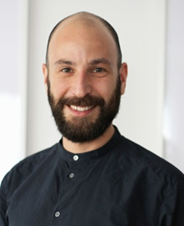 | **H. Günhan Akarçay** completed his MSc at the Ecole Nationale Superieure de Physique de Strasbourg, Strasbourg, France, before joining the Institute of Applied Physics (IAP) of the University of Bern, Switzerland, to work on his PhD until 2012. He then worked as a Postdoctoral Fellow in Montreal, Canada and Ulm, Germany (2012-2013). He is currently Leader of the Light Propagation in Biomedical Applications Group at the IAP. His work focuses on the numerical modeling of light propagation in condensed soft matter and his interests cover radiative transfer, polarimetry, electromagnetism, and statistical physics. |
| 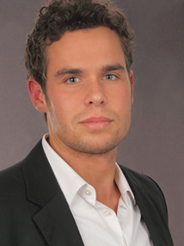 | **K. Gerrit Held** received his M.Sc. degree in Biomedical Engineering from the University of Twente, Enschede, The Netherlands, in 2013 and the PhD degree in Physics from the University of Bern, Bern, Switzerland, in 2017. He is currently carrying out postdoctoral research at the Institute of Applied Physics of the University of Bern, Switzerland. His work focuses on quantitative in vivo imaging of blood oxygenation using a handheld system for combined optoacoustic and ultrasound imaging. |
| 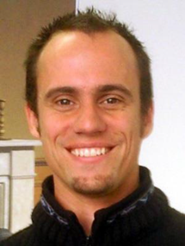 | **Michael Jaeger** received the MSc in Physics from the University of Bern, Bern, Switzerland, in 2002, and the PhD in Physics from the same university in 2007. In 2010/2011, he joined the Institute of Cancer Research and the Royal Marsden Hospital, Sutton, UK. He is group leader of the Optoacoustic Imaging Team at the Institute of Applied Physics, University of Bern, Switzerland. His research interests include optoacoustic imaging with focus on clinical imaging, as well as novel ultrasound techniques such as speed of sound imaging using echo ultrasound. |
| 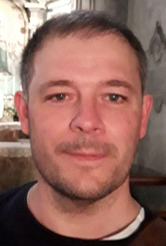 | **Dr. Salvador Sanchez** has a Ph.D. in Particle Physics. He is specialized in photon detectors and in the development of algorithms for Near-infrared Optical Tomography. As a researcher at the University Hospital of Zurich, Switzerland, his scientific activity has been focused on imaging tumor oxygenation and brain ischemic lesions in infants using optical techniques. |
| 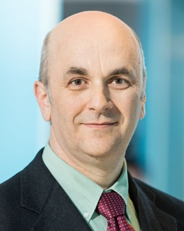 | **Martin Wolf** is professor of Biomedical Optics at the University of Zürich, Zürich, Switzerland. He received his Ph.D. from ETH Zurich. Dr. Wolf heads the Biomedical Optics Research Laboratory, which specializes in developing techniques to measure and quantitatively image oxygenation of brain, muscle, tumor and other tissues. His aim is to translate these techniques to clinical application for the benefit of adult patients and preterm infants. |
| 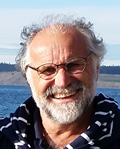 | **Martin Frenz** received his MSc in Physics from the University of Freiburg, Freiburg, Germany, in 1985, and his PhD in Physics from the University of Bern, Bern, Switzerland, in 1990. In 1995, he joined the University of Texas in Austin, USA. In 2002, he became a professor and head of the Biomedical Photonics Department of the Institute of Applied Physics at the University of Bern, Switzerland. Since 2008, he is director of the Institute of Applied Physics. His recent work has explored new imaging modalities in biomedicine, including quantitative optoacoustic imaging and sensing, in vivo microscopy, optoacoustic contrast agents for imaging and therapy, light propagation in tissue as well as laser application for medical diagnostics and therapy. He is a fellow of SPIE and ASLMS. |
